# Persistence of High Levels of Serum Complement C5a in Severe COVID-19 Cases After Hospital Discharge

**DOI:** 10.3389/fimmu.2021.767376

**Published:** 2021-11-18

**Authors:** Yaiza Senent, Susana Inogés, Ascensión López-Díaz de Cerio, Andres Blanco, Arantxa Campo, Francisco Carmona-Torre, Patricia Sunsundegui, Antonio González-Martín, Daniel Ajona, Marcin Okrój, Felipe Prósper, Ruben Pio, José Ramón Yuste, Beatriz Tavira

**Affiliations:** ^1^ Program in Solid Tumors, Translational Oncology Group, Cima-University of Navarra, Pamplona, Spain; ^2^ Department of Biochemistry and Genetics, School of Sciences, University of Navarra, Pamplona, Spain; ^3^ Respiratory Tract Cancer Group, Navarra Institute for Health Research (IdISNA), Pamplona, Spain; ^4^ Department of Immunology and Immunotherapy, Clinica Universidad de Navarra, Pamplona, Spain; ^5^ Area of Cell Therapy and Department of Hematology, Clinica Universidad de Navarra, Pamplona, Spain; ^6^ Department of Internal Medicine, Clinica Universidad de Navarra, Pamplona, Spain; ^7^ Pulmonary Department, Clinica Universidad de Navarra, Pamplona, Spain; ^8^ Division of Infectious Diseases, Clinica Universidad de Navarra, Pamplona, Spain; ^9^ Department of Oncology, Clinica Universidad de Navarra, Madrid, Spain; ^10^ Program in Respiratory Tract Tumors, Centro de Investigación Biomédica en Red de Cáncer (CIBERONC), Madrid, Spain; ^11^ Department of Cell Biology and Immunology, Intercollegiate Faculty of Biotechnology, University of Gdańsk and Medical University of Gdańsk, Gdańsk, Poland; ^12^ Program of Regenerative Medicine, Cima-University of Navarra, Pamplona, Spain; ^13^ Department of Pathology, Anatomy and Physiology, School of Medicine, University of Navarra, Pamplona, Spain

**Keywords:** innate immunity, complement system, C5a, COVID-19, SARS-CoV-2, respiratory symptoms

## Abstract

Evidence supports a role of complement anaphylatoxin C5a in the pathophysiology of COVID-19. However, information about the evolution and impact of C5a levels after hospital discharge is lacking. We analyzed the association between circulating C5a levels and the clinical evolution of hospitalized patients infected with SARS-CoV-2. Serum C5a levels were determined in 32 hospitalized and 17 non-hospitalized patients from Clinica Universidad de Navarra. One hundred and eighty eight serial samples were collected during the hospitalization stay and up to three months during the follow-up. Median C5a levels were 27.71 ng/ml (25th to 75th percentile: 19.35-34.96) for samples collected during hospitalization, *versus* 16.76 ng/ml (12.90-25.08) for samples collected during the follow-up (p<0.001). There was a negative correlation between serum C5a levels and the number of days from symptom onset (p<0.001). C5a levels also correlated with a previously validated clinical risk score (p<0.001), and was associated with the severity of the disease (p<0.001). An overall reduction of C5a levels was observed after hospital discharge. However, elevated C5a levels persisted in those patients with high COVID-19 severity (i.e. those with a longest stay in the hospital), even after months from hospital discharge (p=0.020). Moreover, high C5a levels appeared to be associated with the presence of long-term respiratory symptoms (p=0.004). In conclusion, serum C5a levels remain high in severe cases of COVID-19, and are associated with the presence of respiratory symptoms after hospital discharge. These results may suggest a role for C5a in the long-term effects of COVID-19 infection.

## Introduction

The current outbreak of COVID-19 constitutes a major health challenge worldwide. Most patients with severe acute respiratory syndrome coronavirus 2 (SARS-CoV-2) infection develop none or mild symptoms, but it is estimated that up to 15% of patients progress to severe pneumonia and acute respiratory distress syndrome (ARDS) (1). Severe disease is characterized by an inflammatory response in the viral infected tissue associated with the release of inflammatory cytokines, the recruitment of immune cells, and the activation of coagulation and thrombosis (2, 3). These events originate in the lungs but may extend to other organs, causing a multiorgan damage (4, 5). A better understanding of the immune response that govern the severity of the disease is necessary to understand and clinically manage long-term health consequences of COVID-19.

The complement system represents a major effector of innate immunity against viruses, which mediates potent inflammatory responses (6). A number of studies have shown that deregulated complement activation contributes to the pathogenesis of inflammatory lung diseases (6). Results obtained in preclinical models support a prominent role of the complement system in the pathophysiology of SARS-CoV and other viral infections. In a mouse model of SARS-CoV-1 infection, complement C3 deficiency hampered severe lung pathology and reduced the levels of pro-inflammatory cytokines in association with decrease lung infiltration of neutrophils, monocytes, cytokines and chemokines (7). The anaphylatoxin C5a, a potent immune modulator released from C5 cleavage during complement activation, promotes the recruitment and activation of neutrophils during lung inflammation, which results in acute lung injury and ARDS (8–10). High C5a levels have been described in a range of preclinical models of acute lung injury induced by highly pathogenic viruses, such as SARS-CoV-1, H1N1, H5N1 or H7N9 (8). Moreover, blockade of the C5a/C5a receptor-1 (C5aR1) axis alleviated lung damage in hDPP4-transgenic mice infected with MERS-CoV (11).

Complement over-activation contributes to lung disease in COVID-19 (12). C4d and C5b-9 deposition has been found along the vasculature of the lungs and the skin in hospitalized patients with COVID-19 (13). Expression changes in complement genes were identified in SARS-CoV-2 infected human lung epithelial cells (14). In this study, the interferon-activated JAK1/2-STAT1 signaling pathway and NF-κB were proposed as mediators of intracellular C3 activation in infected cells (14). Another transcriptomic analysis on the peripheral blood of COVID-19 patients revealed an upregulation of C1q and C2 expression in COVID-19 patients, high levels of C5aR1 expression were found in blood and pulmonary myeloid cells, and circulating levels of C5a were associated with the severity of COVID-19 (15). In *in vitro* studies and preclinical models, knock-down or pharmacological inhibition of C5aR1 prevented epithelial destruction (16), and the activation and recruitment of myeloid cells to the lungs (15). C5aR1 blockade also attenuated platelet-mediated COVID-19-associated thrombogenicity in a process dependent on the formation of neutrophil extracellular traps (NETs) (17).

All these data suggest that C5a may be involved in the pathophysiology of COVID-19. Circulating levels of C5a are increased in hospitalized COVID-19 patients as compared with healthy donors (18–21). However, information is lacking on the evolution of C5a levels beyond the hospitalization stay. In the present study, we performed serial measurements of serum C5a levels in COVID-19 patients during the hospitalization stay and after discharge. C5a levels correlated with disease severity, as determined by a previously validated clinical risk score (22). Moreover, C5a levels were associated with the duration and outcome of the hospitalization stay. Interestingly, in those patients with a more severe disease, C5a levels remained elevated weeks after hospital discharge, and were associated with the persistence of respiratory symptoms, suggesting that modulation of complement activation may be an effective therapeutic strategy for the treatment of COVID-19 patients with long-term respiratory problems.

## Materials and Methods

### Patients

Consecutive COVID-19 patients treated at Clinica Universidad de Navarra between April and July 2020 were included in this study. Thirty-two patients were admitted to hospital due to the severity of their symptoms, while 17 individuals, who went to the emergency room due to their symptoms, did not require hospitalization. COVID-19 was diagnosed by SARS-CoV-2 real-time PCR in nasopharyngeal samples. The characteristics of the patients are described in **Table 1**, **Supplementary Table S1**. The study was approved by the Ethics Committee of Clinica Universidad de Navarra (ref. 2020.090), and all patients signed an informed consent.

**Table 1:** Demographic and clinical characteristics of the COVID-19 patients included in the study.

Patient characteristics	n (%)
**Sex**	
Female	28 (57)
Male	21 (43)
**Hospitalization**	
No	17 (35)
Yes	32 (65)
**Comorbidities**	
No	28 (57)
Yes	21 (43)
**Symptoms**	
No	9 (18)
Yes	40 (82)
	**Median (range)**
**Age (years)**	54.5 (45–76)
**COVID risk score**	102 (81–126)
**Laboratory parameters**	
LDH (U/l)	232 (212–312)
D-dimer (ng/ml)	530 (380–1110)
Ferritin (ng/ml)	345 (162–520)
Troponin T (ng/ml)*	6.5 (6.5-14.6)
C-reactive protein (mg/l)	2.04 (0.66-6.65)

*Values below the limit of detection (LOD) were substituted by LOD/2 (6.5 ng/ml).

### Serum Collection

Venous blood samples were collected in BD Vacutainer CAT tubes (ref. 367896) periodically during the hospital stay, and up to three months after being discharged from the hospital. Blood samples were immediately processed after collection. Serum was obtained by centrifugation at 3,500 rpm for 8 minutes and stored at -80°C. Those samples collected during hospitalization were classified as H1 (for samples collected between 1 and 3 days after admission; n = 24), H5 (4 to 5 days; n = 25), H8 (7 to 9 days, n = 22), and H14 (11 to 15 days; n = 9). Follow-up samples collected after hospital discharge, or from non-hospitalized patients, were categorized as F7 (ranging from 5 to 10 days after discharge; n = 29), F14 (11 to 23 days; n = 39) and F90 (69 to 123 days; n = 40).

### Marker Quantification

C5a was quantified in serum samples using Luminex technology and the anti-C5a capture and detection antibodies from the Human Complement Component C5a DuoSet ELISA kit (DY2037, R&D Systems). C5b-9 levels, routine laboratory markers and pro-inflammatory cytokines IFNγ and IL1α were also determined. Details of the procedures are provided in **Supplementary Methods**. Analyses were performed blindly.

### Statistical Analyses

Normal distribution of data was assessed by the Shapiro-Wilk test, and the density and q-q plots. Non-parametric Mann-Whitney *U* and Kruskal-Wallis tests were applied to compare two or more groups, respectively. Spearman’s rank coefficient was used to measure the correlation between continuous variables. Pearson Chi-square was used to evaluate differences between categorical variables. Continuous variables were described as median (lower to upper quartile). All tests were two-sided, and a p value less than 0.05 was considered statistically significant, with *p < 0.05, **p < 0.01 and ***p < 0.001. R v4.0.2 (R Core Team, Vienna, Austria, 2020) was used for statistical analysis. Figures were produced with the R packages ggplot2 (23) and ggpubr (24). The R package tidyverse was used for data manipulation (25).

## Results

### Characteristics of COVID-19 Patients

This study included patients who came to Clinica Universidad de Navarra presenting symptoms compatible with COVID-19 and were hospitalized or not due to the severity of these symptoms (n = 32 and n = 17, respectively). SARS-CoV-2 infection was diagnosed by real-time PCR. Demographic characteristics, co-morbidities symptoms and routine are shown in **Table 1**, **Supplementary Table S1**. Patients had a median age of 54 (25^th^ to 75^th^ percentile: 45-76) years. Gender distribution was 57% females and 43% males. Several comorbidities were diagnosed, such as diabetes (8%), hypertension (31%), cardiopathy (24%), renal insufficiency (8%) or obesity (37%). Men showed higher frequencies of hypertension (p = 0.011) and cardiopathy (p = 0.003). More than half of the patients showed fever (55%) and/or coughing (63%). Regarding SARS-CoV-2 symptoms, no gender differences were found.

Patients were stratified into five COVID severity groups according to the number of hospitalization days (low, medium or high severity), or whether they were not hospitalized (very low severity) or died during hospitalization (very high severity). No patient in our cohort required UCI admission. Low severity was considered when the hospitalization period was equal or lower than 7 days, medium when it was between 8 and 13 days, and high if it was equal or higher than 14 days. A more detailed description of the distribution of hospitalization days per group is shown in **Supplementary Table S2**. Age was significantly associated with severity: 42 (35–47), 56 (48–58), 54 (44–76), 84 (75–87) and 88 (83–92) years for very low, low, medium, high or very high severity groups, respectively (p < 0.001). The presence of comorbidities (p = 0.022), higher frequencies of some them, such as diabetes (p = 0.014) or cardiopathies (p = 0.024), or the presence of respiratory problems (p = 0.037) were also associated with the severity of the disease. Disease severity was also associated with the basal levels of laboratory markers known to be associated with COVID-19-related complications, such as lactate dehydrogenase (LDH; p = 0.017), D-dimer (p = 0.004), troponin T (p < 0.001) or C-reactive protein (CRP; p = 0.039). Viral load at admission was not associated with disease severity (p = 0.860 for E gene expression, and p = 0.740 for N gene expression).

A clinical risk score, which estimates the risk of developing critical illness among hospitalized COVID-19 patients, was calculated for hospitalized patients in our cohort. The score is based on ten variables commonly measured on admission to the hospital: abnormal chest radiography findings, age, dyspnea, hemoptysis, unconsciousness, number of comorbidities, cancer history, neutrophil-to-lymphocyte ratio, lactate dehydrogenase, and direct bilirubin (22). The median risk score was 119, with an interquartile range of 88 to 142, and minimum and maximum values of 54 and 209, respectively. The risk score was associated with the severity of the disease: 82 (80–100), 116 (90–121), 138 (134–147) and 191 (191–205) for low, medium, high and very high severity groups, respectively (p < 0.001).

### Serum C5a Levels in COVID-19 Patients

Serum samples were collected periodically from hospitalized patients during their hospital stay and at different time points after hospital discharge. Longitudinal samples from non-hospitalized patients were also collected during the follow-up of the patients. The follow-up days for hospitalized patients ranged from 14 to 106 days, with a median of 92 (81–97) days. In the case of non-hospitalized patients, the follow-up days ranged from 14 to 123 days, with a median of 106 (96–109) days. One hundred and eighty eight serum samples were collected (80 at hospitalization and 108 at follow-up). The median of serum C5a levels was 20.45 ng/ml, with an interquartile range of 14.66 to 30.23 ng/ml and minimum and maximum values of 6.11 and 57.18 ng/ml, respectively. C5a levels along the different time points in each patient are shown in **Supplementary Figure S1**. A negative correlation (ρ = -0.38, p < 0.001) was found between C5a levels and the number of days from symptom onset at sample collection (**Supplementary Figure S2A**). Besides, C5a levels in samples collected during hospitalization were significantly higher than in samples collected during follow-up (27.71 [19.35-34.96] *vs*. 16.76 [12.90-25.08]; p < 0.001; **Supplementary Figure S2B**). Viral load at admission was not associated with C5a levels (ρ = -0.08, p = 0.616, for E gene expression; and ρ = -0.17, p = 0.302, for N gene expression). As expected, C5a levels correlated with C5b-9 levels (ρ = 0.54, p < 0.001). C5a levels also correlated with basal levels of LDH (ρ = 0.38, p = 0.008), D-dimer (ρ = 0.39, p = 0.006), ferritin (ρ = 0.54, p < 0.001), troponin T (ρ = 0.37, p = 0.008) and CRP (ρ = 0.43, p = 0.002). Finally, basal levels of pro-inflammatory makers IFNγ and IL1α could be assessed in 25 hospitalized patients. C5a serum levels significantly correlated with IL1α levels, but not with IFNγ levels (ρ = 0.75, p < 0.001 and ρ = -0.19, p = 0.373, respectively). All these correlations are shown in **Supplementary Figure S3**.

### Serum C5a and COVID-19 Severity During Hospitalization and Follow-up

**Figure 1:**
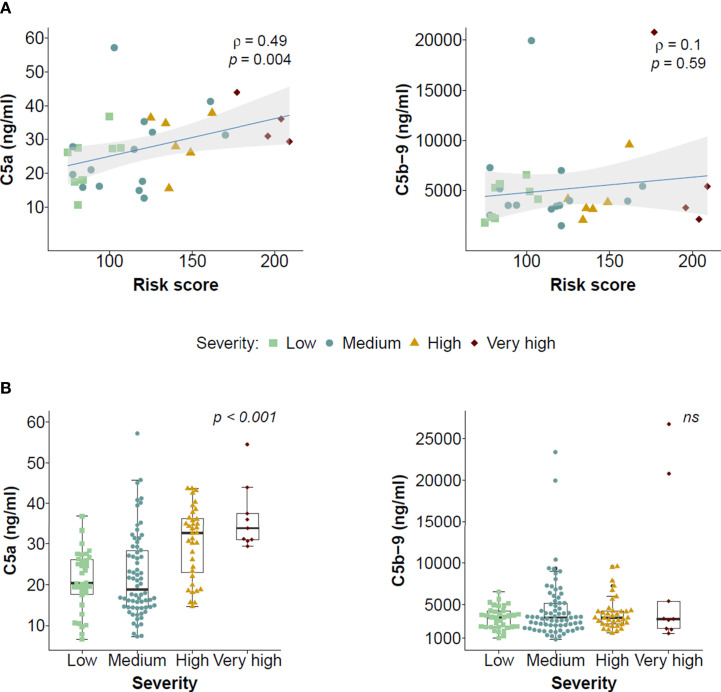
Association of serum levels of C5a and C5b-9 with disease severity outcome in hospitalized COVID-19 patients. **(A)** Correlation between the levels of C5a or C5b-9 in the first serum sample collected after hospitalization and a clinical risk score calculated for each patient. The correlation coefficients, the statistical significances of the Spearman’s test and the 95% confidence intervals (shadowed areas) are shown. The severity group at which each patient was assigned is also shown. **(B)** Association between serum C5a and C5b-9 levels in the first serum sample collected after hospitalization and severity of the disease. Statistical differences were analyzed using the Kruskal-Wallis test.

C5a levels in the first sample collected at hospitalization correlated with the clinical risk score assigned to that patient: ρ = 0.49, p = 0.004 (**Figure 1A**). Moreover, those hospitalized patients with a more severe disease outcome showed higher serum C5a levels (p < 0.001; **Figure 1B**). Interestingly, this correlation was not observed for the other complement-related marker. Thus, C5b-9 levels did not correlate with the risk score (ρ = 0.10, p = 0.590), and were not associated with clinical outcome (p = 0.850). No other remarkable association was found between C5a levels and the clinicopathological characteristics of the patients (**Supplementary Table S3**).

**Figure 2:**
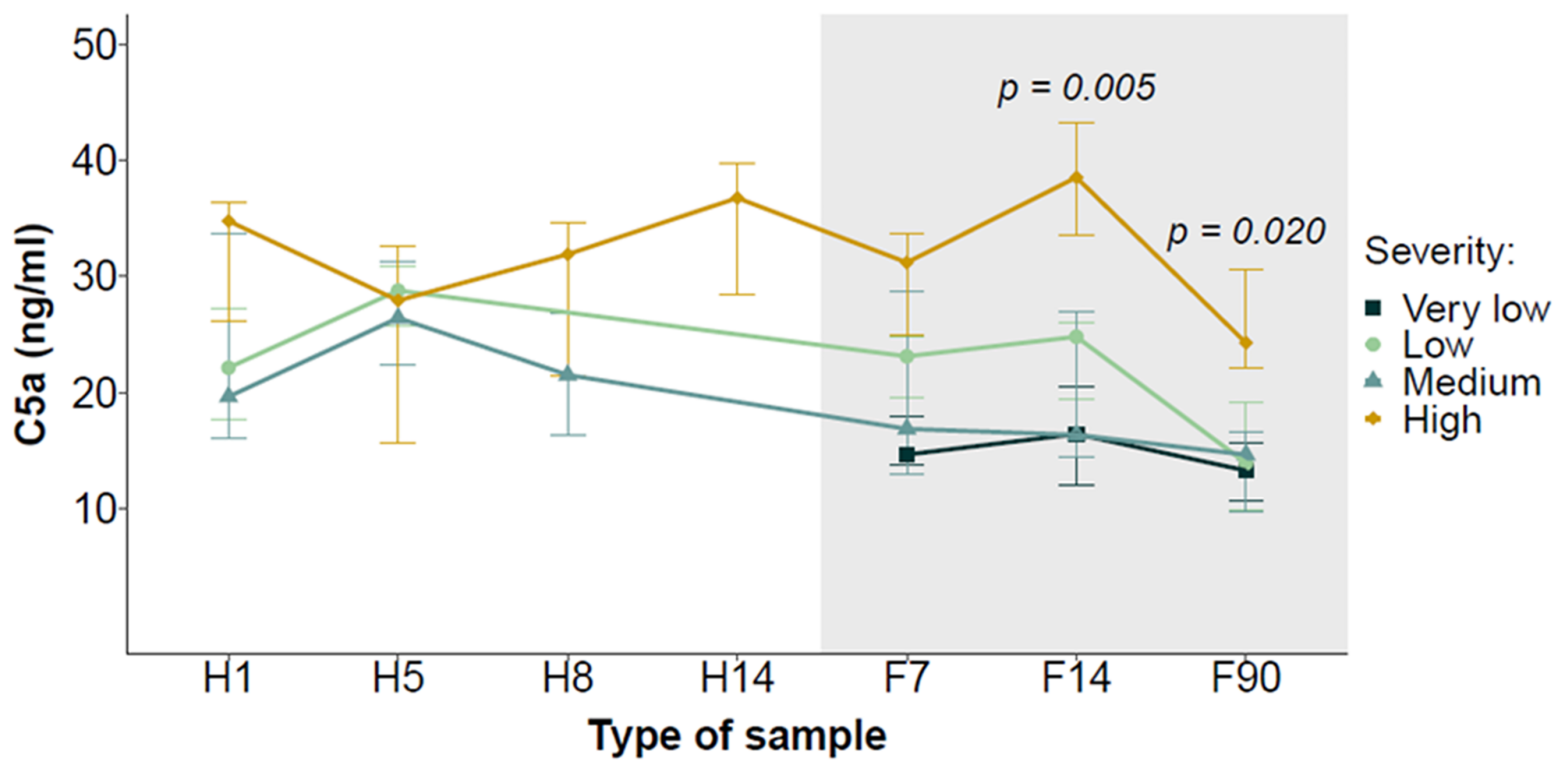
Evolution of serum C5a levels during hospitalization and follow-up in COVID-19 patients stratified by the severity of the disease. Serial samples were collected at different time points during the hospitalization period (H1, H5, H8 and H14) and the follow-up (F7, F14 and F90). Data are presented as median ± interquartile range. The shadowed area corresponds to the follow-up period. Differences between groups at each time point were analyzed using the Kruskal-Wallis test.

To monitor the evolution of C5a levels, as detailed in Material and Methods, samples were categorized as H1, H5, H8 and H14 for those samples collected during hospital stay, and as F7, F14 and F90 for those samples collected after hospital discharge or from non-hospitalized patients. Patients who died during their hospital stay were excluded from this part of the study since they did not have follow-up samples. Persistently elevated C5a levels were observed in those patients with a longest stay in the hospital (**Figure 2**). C5a levels in patients with low or medium COVID-19 severity were similar to those in non-hospitalized patients as soon as one week after hospital discharge. The last time point of follow-up (F90) revealed a trend to decreasing C5a levels, suggesting that marker levels begin to normalize at this time point. The same trend was observed when hospitalized patients were stratified as high or low risk according to the clinical risk score (**Supplementary Figure S4**). These results suggest that complement C5a levels in serum samples collected from COVID-19 patients remain high and are associated with the severity of the disease weeks after hospital discharge.

### Serum C5a and Respiratory Problems in COVID-19 Patients After Discharge

**Figure 3:**
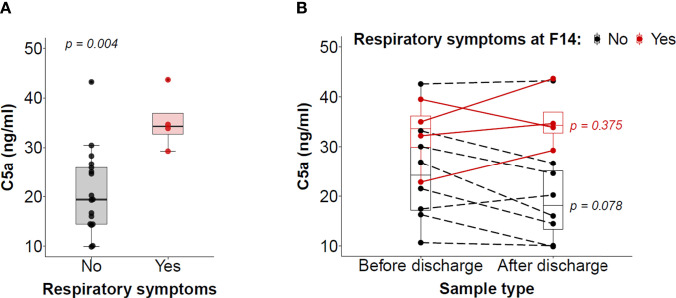
Association between serum C5a levels and the presence of respiratory symptoms. **(A)** Association between serum C5a levels and the presence or not of respiratory symptoms two weeks after hospital discharge. **(B)** Evolution of serum C5a levels from the last sample at hospitalization and the follow-up sample at F14 in patients who suffered respiratory problems during their hospitalization. Patients are divided into patients with or without respiratory problems in the follow-up visit (red and black, respectively). Differences were analyzed with the Wilcoxon signed-rank test for paired samples.

We sought to assess whether there was an association between serum C5a levels and persistent health problems in those patients discharged from the hospital after recovery from COVID-19. Symptom data were recorded during the follow-up visits at F14 and F90 in 25 and 24 discharged patients, respectively (**Supplementary Table S4**). Musculoskeletal (arthralgia and fatigue) and respiratory symptoms (cough and dyspnea) were the most frequent health problems found in the patients. At F14, no association was found between C5a levels and musculoskeletal problems. However, significantly higher C5a levels were found in patients with respiratory symptoms as compared with those in patients without respiratory symptoms (p = 0.004; **Figure 3A**). Moreover, C5a levels during hospitalization may predict which patients will present persistent respiratory symptoms after hospital discharge, as shown by the receiver operating characteristic (ROC) curve generated using C5a levels at H8 and respiratory symptoms recorded at F14 (AUC: 0.846 [0.650-1.000]; **Supplementary Figure S5**). Nevertheless, this result should be considered preliminary because only four patients showed respiratory problems at this follow-up time point. No analysis could be performed at F90 due to the scarcity of discharged patients with respiratory symptoms (e.g. only one patient with mild dyspnea).

We next evaluated the changes in C5a levels between the samples collected before hospital discharge and the samples collected at F14 in those hospitalized patients who had respiratory problems. While not all patients followed the same evolution, an overall reduction in C5a levels was observed in those patients in whom the respiratory symptoms had disappeared at F14 (p = 0.078), but not in those patients who still were experiencing respiratory symptoms (**Figure 3B**). Finally, at F90, respiratory symptoms had disappeared in two patients with respiratory problems at F14. In both cases, C5a levels decreased between the two follow-up time points (from 29.25 to 16.91 ng/ml, and from 43.65 to 30.62 ng/ml). These results suggest that elevated C5a levels in circulation may be associated with the persistence of respiratory problems in patients discharged from the hospital after recovery from COVID-19.

## Discussion

In this study, we report the persistent presence of high levels of circulating complement C5a, a cleavage product of terminal complement C5, in COVID-19 patients with a long hospital stay, even weeks after hospital discharge. Our data also suggest that C5a is linked to the persistence of respiratory symptoms after hospital discharge. Long-term health consequences have been reported in 50 to 80% of patients discharged from the hospital after recovery from COVID-19 (26–28). Patients who are more severely ill during their hospital stay are especially susceptible to suffer for impaired pulmonary function (29), and are considered the main target population for intervention (30). However, the biological mediators responsible for the pathophysiology of long-term symptoms are mostly unknown.

Circulating levels of C5a are increased in hospitalized COVID-19 patients as compared with healthy donors (18–21), while this does not appear to be the case in non-hospitalized patients (20). About the association between C5a levels and the severity of the disease in hospitalized individuals, Carvelli et al. found higher plasma C5a levels in those hospitalized patients with lung damage (15). C5a levels were higher in severe COVID-19 patients who required intensive care unit admission (21). In another study performed with patients at intensive care unit, C5a correlated with hypoxemia (determined as PaO_2_/FiO_2_ ratio) at the time of admission (31). In contrast, in other studies, elevated C5a levels were found in hospitalized patients regardless of the severity of the disease (18, 19). The presence of a genetic variant linked to severe COVID-19 predisposition (rs11385942) was associated with increased circulating C5a levels on the day of admission, but these levels did not correlate with markers of inflammation or tissue damage (32). Patients with respiratory failure, either at admission or developed during hospital stay, showed significantly higher levels of soluble C5b-9 and C4d, but not of C5a (33). In our study, at the time of admission, we did not find any association between C5a levels and COVID-related symptoms. However, those patients who were going to require a longer hospital stay showed higher C5a levels. C5a levels at admission also correlated with a previously reported score that estimates the risk of developing critical illness among hospitalized COVID-19 patients (22), and with several laboratory values known to be associated with COVID-19 severity. These results suggest that the effects mediated by C5a become more evident over the course of the disease. Only in those patients in which high C5a levels persist, or are further induced, this molecule may exert more clearly its influence. In this line, in hospitalized COVID-19 patients on hemodialysis, clinical deterioration was preceded by a peak of C5a levels, suggesting that C5a may be a marker of disease progression (20). In a study that monitored the evolution of C5a levels during the hospitalization stay, in those patients who stayed less than 15 days in the hospital, C5a levels decreased from admission to day 10, whereas this reduction was not observed in patients with longer hospitalization (18). We also observed that C5a levels were maintained in those patients with a longest stay in the hospital. On the other hand, until now very little information existed about the evolution of C5a levels after hospital discharge. Cugno et al. reported a significant reduction of serum C5a levels after discharge (19). We also report now an overall reduction of C5a at the follow-up visits, with levels comparable to those from non-hospitalized patients. However, this reduction was not observed in those patients with a longer hospital stay, in whom C5a remained high in association with persistent respiratory symptoms.

Activation of different complement pathways may be responsible for the release of C5a during the acute phase of COVID-19. Holter et al. found a weak correlation between antibodies against SARS-CoV-2 and complement activation in hospitalized patients (33). Deposits of C4d, mannose binding lectin-associated serine protease-2 (MASP-2) and C5b-9 were found in the lung microvasculature of patients with severe COVID-19 (13). Ma et al. found that factor D strongly correlates with markers of endothelial injury and coagulation (21). Therefore, there is evidence for the implication of the three major pathways of complement activation in COVID-19 pathogenesis. Sustained high C5a levels implies a state of persistent production of the peptide from C5, since C5a has a very short half-life *in vivo* (it is readily captured by its receptors, or rapidly inactivated by carboxypeptidases). Interestingly, although we found a significant correlation between the levels of C5a and the terminal complement complex C5b-9, the latter marker was not associated with the clinical risk score or the severity of the disease. This result suggests the existence of alternative mechanisms of C5 activation that may be governing the long-lasting production of C5a levels. Noteworthy, C5a is not only produced by the canonical C5 convertase, but also by extrinsic factors such as thrombin (34). Since an enhanced thrombin-generating capacity has been observed in COVID-19 patients after months from hospital discharge (35), we can speculate that persistent C5a production may be caused by this abnormal hemostatic state. An unresolved prothrombotic state, maybe associated with dysfunction of the endothelium, may enhance thrombin production, which, subsequently, would lead to the cleavage of C5 into C5a. The implication of other potential mechanisms, such as a prolonged viral shedding that would maintain complement activation or a chronic state of unresolved immune response, also merit further investigation.

Growing evidence supports the role played by C5a in the pathogenesis of respiratory distress following SARS-CoV-2 infection (36, 37). C5a orchestrates a strong inflammatory response (38), and is a potent mediator of the acute lung injury induced by viral infections (8–11). C5aR1 blockade with monoclonal antibodies prevented C5a-mediated recruitment and activation of human myeloid cells, and inhibited acute lung injury in human C5aR1 knock-in mice (15). Stimulation of myeloid cells by C5a also contributes to thrombosis and tissue damage (17, 39). Excessive activation of the complement cascade, e.g. by antibodies against SARS-CoV-2 (30) or the activation of the lectin pathway (12), may lead to an over-production of C5a, resulting in cytokine storm, severe lung inflammation, infiltration of immune cells, endothelial dysfunction and thromboinflammation (40, 41). Elevated C5a levels may sustain endothelial dysfunction and an unresolved prothrombotic state, creating a vicious circle that would perpetuate the respiratory problems observed in some discharged patients. If this holds true, patients with long-term respiratory problems would benefit from therapies targeting C5a production.

From the early onset of the pandemic, complement intervention to decrease C5a levels was proposed as an anti-inflammatory strategy that may overt the excessive inflammatory response seen in severe cases of COVID-19 (36, 42). Results from early clinical studies targeting complement at the level of C3 or C5 have already been reported (15, 43–48). They included the use of avdoralimab (IPH 5401), a recombinant human monoclonal antibody that targets C5aR1; AMY-101, a peptidic C3 inhibitor; eculizumab, a humanized monoclonal antibody that inhibits C5 cleavage; or vilobelimab (IFX-1), a chimeric monoclonal antibody that binds to C5a. Some preliminary data have been obtained, showing tolerable adverse effects and potential clinical benefit associated with an improvement in respiratory function (46). While both C3 and C5 inhibition lead to sustained anti-inflammatory response, it has been suggested that C3 inhibition may have a more prominent effect on endothelial or alveolar injury (47). Results from larger controlled trials are awaiting to confirm the clinical efficacy of complement intervention, and to identify the best inhibitory strategy. Given the cost, it may be also necessary to select those patients that more likely would benefit from these treatments by using circulating markers or genetic variants linked to severe COVID-19 predisposition.

We acknowledge some limitations in our study. We have used serum samples, whereas plasma is the preferred sample type for the determination of complement factors. The number of patients included in the study is limited, which may have reduced the ability to detect differences between groups. It also limited our capacity to evaluate the diagnostic performance of C5a as a predictive marker for persistence respiratory problems. Besides, the study was conducted with participants recruited in a single center. For all these reasons, validation studies in independent cohorts are required for a generalization of our results, and for exploring their clinical application. More experiments are also required to assess if C5a levels are specific of persistent respiratory symptoms after COVID-19 infection or constitute a common feature of many persistent respiratory diseases. The correlation observed between C5a and IL1α serum levels also merit further research.

In conclusion, we report for the first time the persistence of elevated serum C5a levels in discharged COVID-19 patients who suffered a severe manifestation of the disease. Moreover, sustained high C5a levels may be associated with long-term respiratory symptoms. Complement activation may be considered as a therapeutic target for the treatment of severe COVID-19 patients with persistent respiratory complications, at least in those cases in which there is an excessive complement activation.

## Data Availability Statement

The raw data supporting the conclusions of this article will be made available by the authors, without undue reservation.

## Ethics Statement

The study was approved by the Ethics Committee of Clinica Universidad de Navarra (ref. 2020.090). The patients provided their written informed consent to participate in this study.

## Author Contributions

YS, RP, JY, and BT conceptualized the study and supervised the work. SI, AL-DC, AB, AC, FC-T, PS, FP, and JY were responsible for the collection of clinical samples, sample processing and/or data management. YS, MO, and BT carried out the experiments. YS, AG-M, JY, RP, and BT performed the analyses. YS, DA, RP, and BT wrote the first draft of the manuscript. All authors contributed to the article and approved the submitted version.

## Funding

This work was supported by Foundation for Applied Medical Research (FIMA), Fundación MTorres, Fundación Ramón Areces, Fondo de Investigación Sanitaria-Fondo Europeo de Desarrollo Regional “Una manera de hacer Europa” (PI20/00419), Departamento de Salud de Gobierno de Navarra (0011-3638-2020-000004), and Departamento de Desarrollo Económico y Empresarial del Gobierno de Navarra (AGATA and DESCARThES projects).

## Conflict of Interest

The authors declare that the research was conducted in the absence of any commercial or financial relationships that could be construed as a potential conflict of interest.

## Publisher’s Note

All claims expressed in this article are solely those of the authors and do not necessarily represent those of their affiliated organizations, or those of the publisher, the editors and the reviewers. Any product that may be evaluated in this article, or claim that may be made by its manufacturer, is not guaranteed or endorsed by the publisher.
