# Supplementary material for: Persistence of High Levels of Serum Complement C5a in Severe COVID-19 Cases After Hospital Discharge
Source: Front Immunol. 2021 Nov 18;12:767376. doi: 10.3389/fimmu.2021.767376 (PMC8636747; doi:10.3389/fimmu.2021.767376)
Supplement: Supplementary file 1 [file DataSheet_1.pdf]

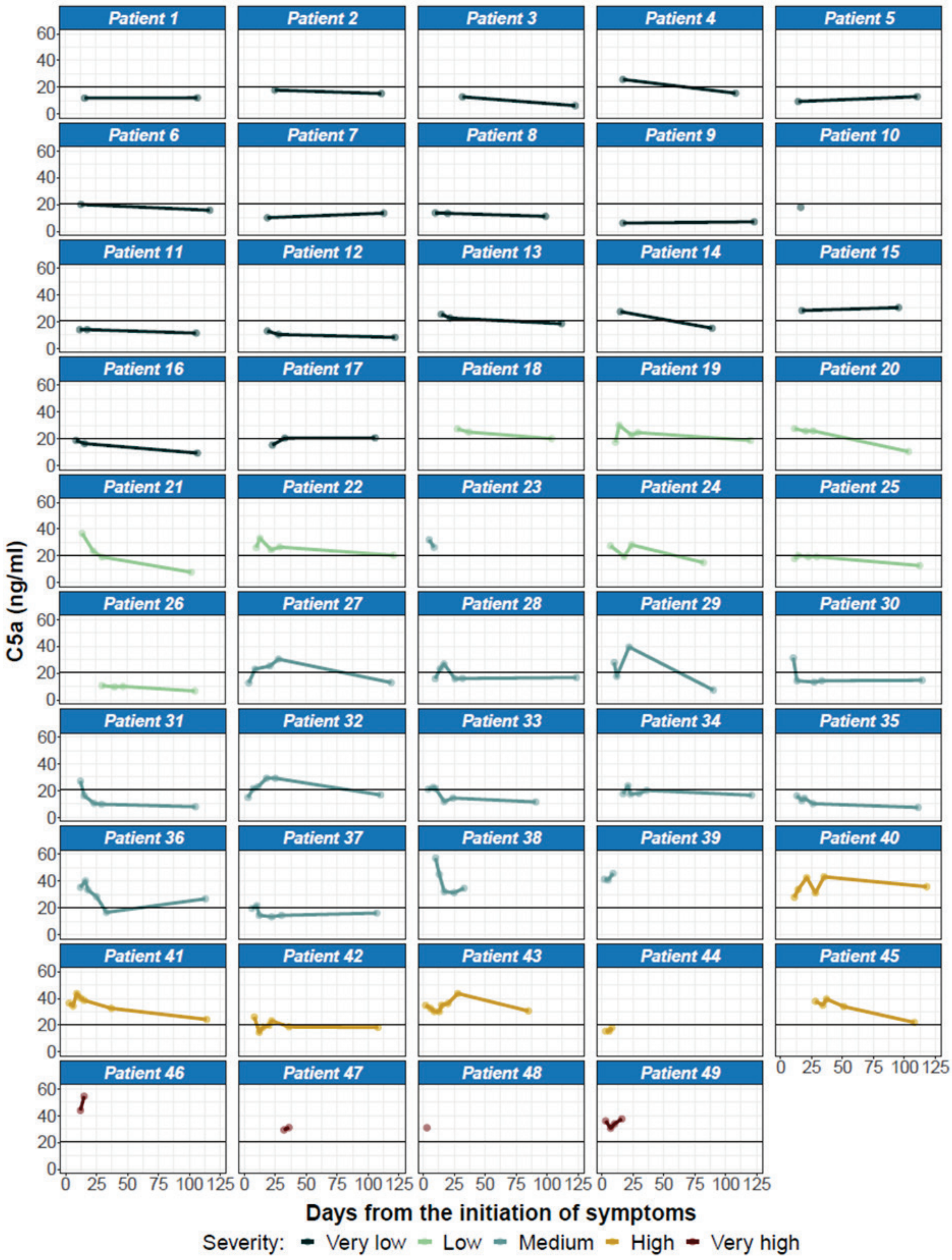

Supplementary Figure S1. Longitudinal follow-up of serum C5a levels in non-hospitalized and hospitalized COVID-19 patients. C5a levels (ng/ml) are presented according to the number of days since the onset of symptoms. Patients were stratified in severity groups based on the number of hospitalization days. The horizontal bar in each graph represents the median value of C5a in the cohort.

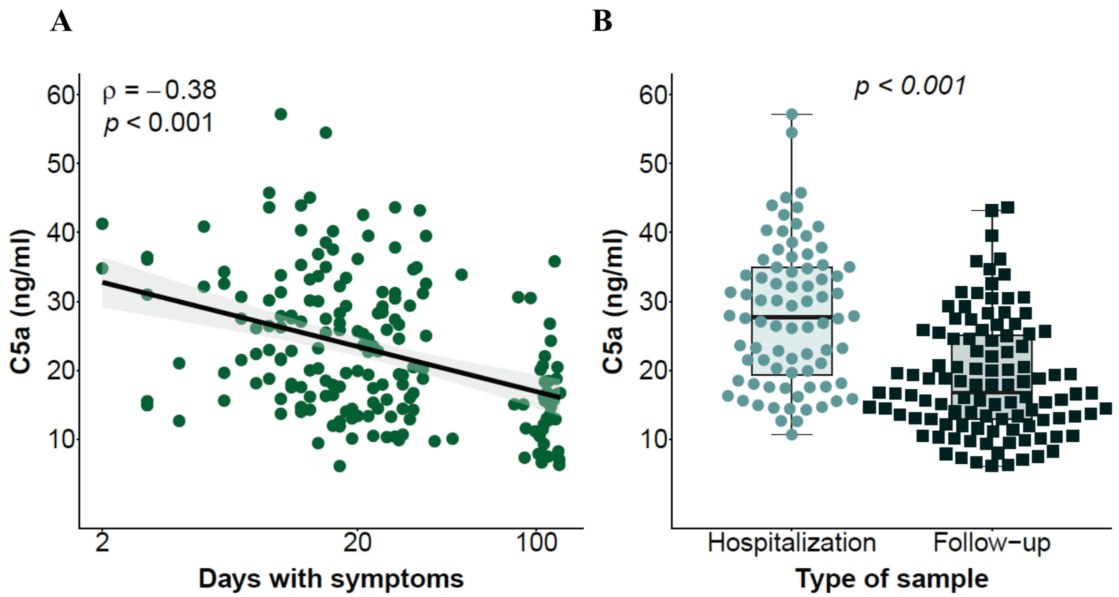

Supplementary Figure S2. A. Correlation between circulating levels of C5a in COVID-19 patients and the days from symptom onset at sample collection. The correlation coefficient and the statistical significance of the Spearman's test are shown. **B.** Association between circulating C5a levels and sample type (samples collected during hospitalization or samples collected during the follow-up of the patients).

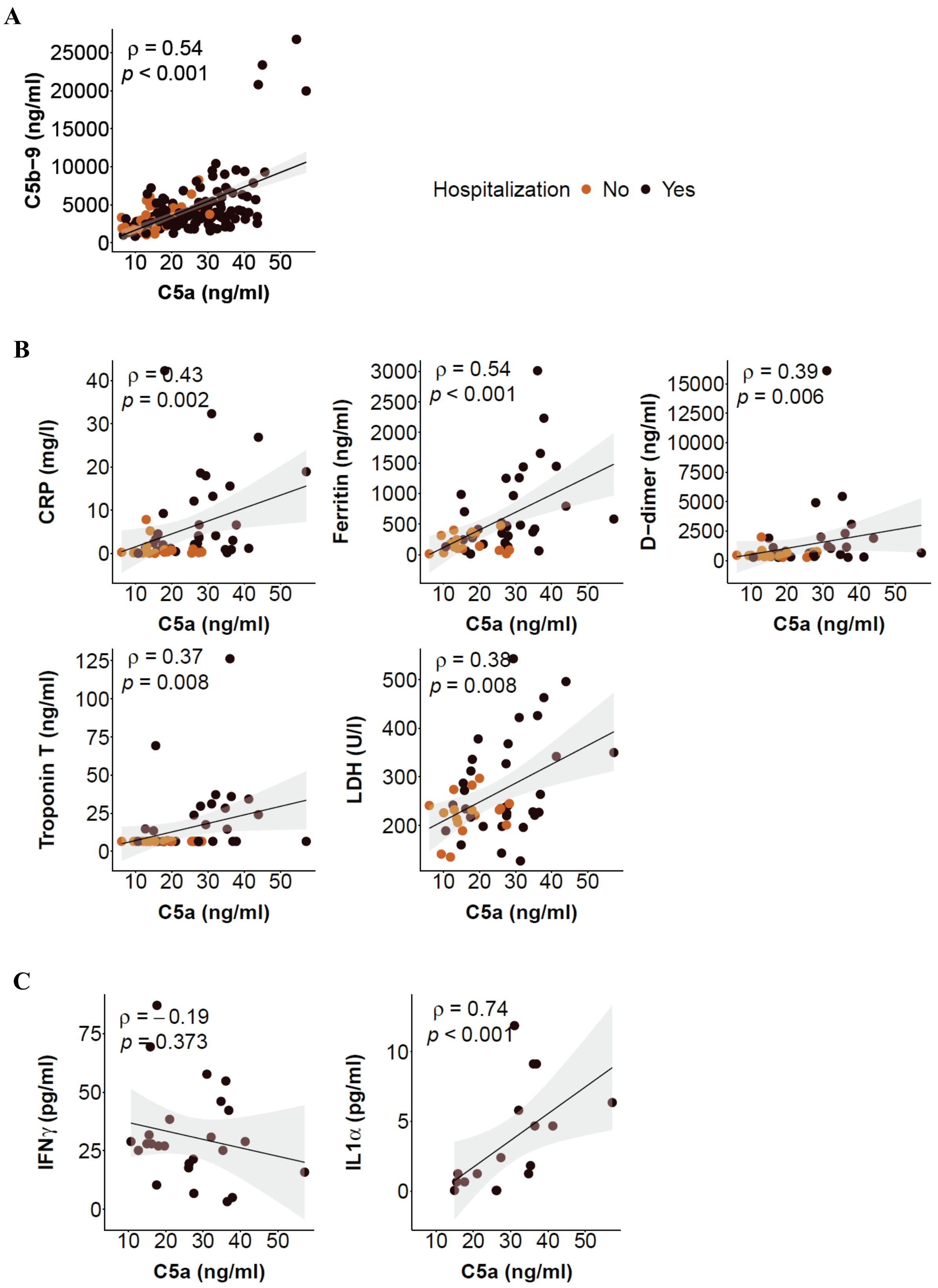

Supplementary Figure S3. Correlations between C5a levels and levels of complement C5b-9 (A), some laboratory markers associated with COVID-19 severity (B) and pro-inflammatory cytokines IFNγ and IL1α (C) in serum samples from COVID-19 patients. The correlation coefficient and the statistical significance of the Spearman's test are shown. Whether the patient was hospitalized or not is also indicated.

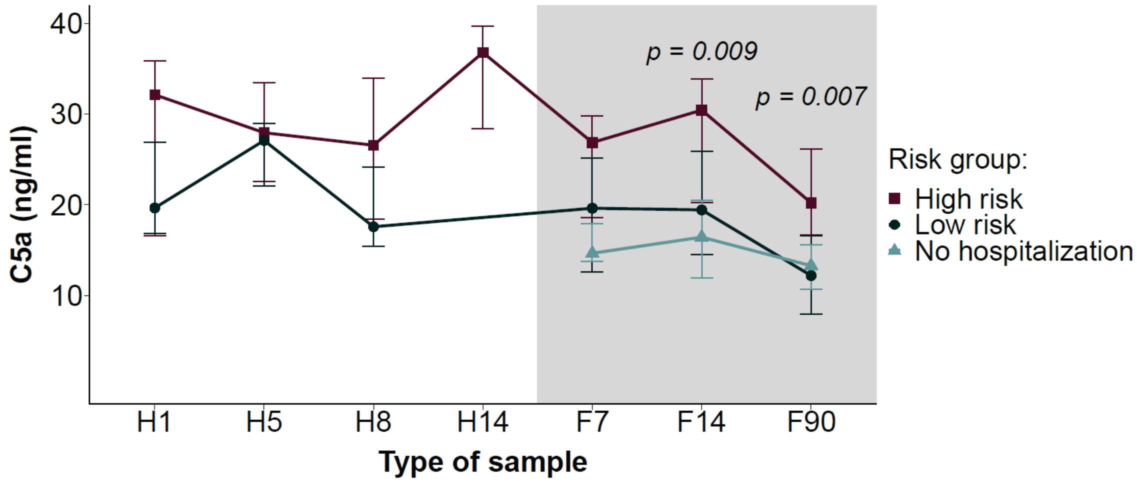

Supplementary Figure S4. Evolution of serum C5a levels during hospitalization and follow-up in COVID-19 patients stratified by the clinical risk score as high or low risk based on the median value of the variable. C5a levels in the follow-up samples from non-hospitalized patients are also shown. Serial samples were collected at different time points during the hospitalization period (H1, H5, H8 and H14) and the follow-up (F7, F14 and F90). Data are presented as median ± interquartile range. The shadowed area corresponds to the follow-up period. Differences between groups at each time point were analyzed using the Kruskal-Wallis test.

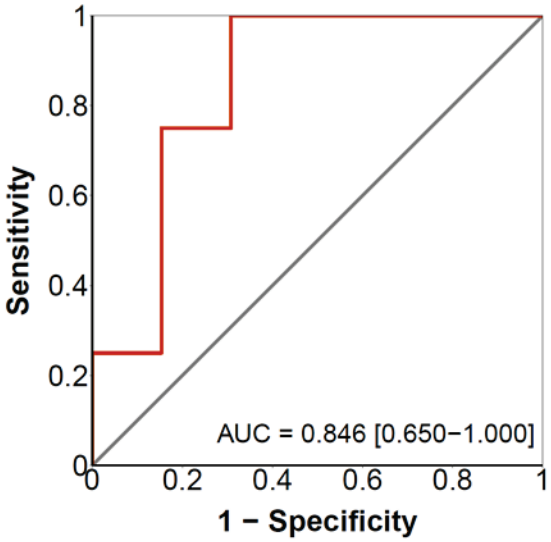

Supplementary Figure S5. ROC curve for the performance of C5a serum levels as predictors of respiratory problems after hospital discharge. C5a values collected at the H8 hospitalization time point (7 to 9 days after admission) were used. Respiratory problems were assessed at the F14 follow-up time point (11 to 23 days from discharge). At F14, the number of cases without and with respiratory symptoms was 13 and 4, respectively. The AUC of the ROC curve is also shown.
