# Supplementary material for: Persistence of High Levels of Serum Complement C5a in Severe COVID-19 Cases After Hospital Discharge
Source: Front Immunol. 2021 Nov 18;12:767376. doi: 10.3389/fimmu.2021.767376 (PMC8636747; doi:10.3389/fimmu.2021.767376)
Supplement: Supplementary file 2 [file DataSheet_2.pdf]

SUPPLEMENTARY METHODS

C5a quantification

Luminex magnetic microspheres were coated with the capture anti-C5a antibody provided in the Human Complement Component C5a DuoSet ELISA kit (DY2037, RD Systems). Luminex MagPlex microspheres (region 12, MC10012-01, Luminex) compatible with Magpix instrument were used. The xMAP Antibody Coupling (AbC) kit (40-50016, Luminex) was used to covalently couple the anti-C5a antibody to 12.5 million carboxylated magnetics beads. Magnetic beads were stored protected from light at 4ºC.

Serum samples from patients were diluted 1:20 for a total volume of 50 µl using working buffer (PBS supplemented with 1% BSA and 0.05% sodium azide). Washing buffer contained PBS + 0.05% Tween 20. All incubations were performed at room temperature in a 96-well plate (Greiner Bio-One 655096) protected from light. Fifty µl of beads, standards and samples were mixed and incubated for 2 hours at RT. Afterwards, the plate was attached to a magnetic plate for 2 minutes and washed. This step was repeated twice. The detection antibody against C5a (from the Human Complement Component C5a DuoSet ELISA kit) was diluted in working buffer 1:1,000 and 100 µl were dispensed per well. Streptavidin-PE was added in the same proportion (1:1) after one-hour. In the last step, magnetic microspheres were resuspended in 150 µl of working buffer by shaking 5 min at 700 rpm. Mean fluorescence intensity (MFI) per sample was determined in a Magpix instrument with xPONENT software (Settings: Type of analysis = None). MFIs were transformed into concentration values (ng/ml) by using a reference curve obtained with the C5a standard provided in the R&D kit. Inner controls between plates were used to calculate a correlation factor applied to data normalization.

C5b-9 quantification

ELISA plates (Maxisorp, Nunc) were coated with 5 µg/ml of mouse monoclonal anti-C5b-9 neoepitope antibody (clone ae11, Hycult) overnight in PBS buffer at 4ºC, and blocked with 1% fish gelatin solution (Sigma Aldrich) in washing buffer (20 mM Tris, 140 mM NaCl, 0.2% Tween 20) for 1 hour at room temperature. Serum samples were diluted 1:40 in working buffer (PBS supplemented with 0.2% Tween 20 and 20 mM EDTA), and added to the plate. The standard curve was prepared from purified C5b-9 complex (Complement Technology) diluted in 1:40 normal human serum in working solution. After 1 h incubation at 4ºC, plates were washed, incubated for 45 min at 37ºC with a rabbit anti-C5b-9 neo polyclonal antibody (Complement Technology) diluted 1:5,000 in PBS + 0.2% Tween 20, followed by washing and 30 min incubation with 1:2,500 dilution of goat-anti-rabbit HRP conjugate (Jackson Laboratory). The assay was developed with TMB solution (Sigma) and stopped with 0.5 M H_2SO_4. Absorbance at 450 nm was measured with Synergy H1 reader (BioTek), and C5b9 concentration in samples vs. the standard curve was determined using GraphPad Prism software.

Routine laboratory tests and cytokine determinations

Routine laboratory parameters (LDH, ferritin, D-dimer, CRP and troponin T) and cytokines (IFNγ and IL1α) were determined following standard procedures at the Department of Clinical Chemistry, Clínica Universidad de Navarra, Pamplona, Spain.
