# Supplementary material for: Persistence of High Levels of Serum Complement C5a in Severe COVID-19 Cases After Hospital Discharge
Source: Front Immunol. 2021 Nov 18;12:767376. doi: 10.3389/fimmu.2021.767376 (PMC8636747; doi:10.3389/fimmu.2021.767376)
Supplement: Supplementary file 3 [file DataSheet_3.pdf]

Supplementary Table S1. Detailed co-morbidities and symptoms in the COVID-19 patients included in the study

	n (%)
**Comorbilities**	
Hypertension	15 (31)
Diabetes	4 (8)
Cardiopathy	12 (24)
Kidney disease	5 (10)
COPD	5 (10)
Immunosupression	4 (8)
Cancer	3 (6)
Obesity	18 (36)
Overweight	13 (26)
Obese	5 (10)
**Symptoms**	
Fever	27 (55)
Malaise	13 (26)
Arthromyalgia	12 (24)
Asthenia	11 (22)
Respiratory problems	34 (70)
Cough	31 (63)
Dyspnea	16 (33)
Digestive problems	12 (24)
Nausea	7 (14)
Diarrhea	9 (18)
Neurologic problems	11 (22)
Headhache	8 (16)
Anosmia	4 (8)
Ageusia	4 (8)

Supplementary Table S2. Summary of the hospitalization days included in each of the COVID-19 severity groups

Severity	n	Median	Min.	Max.
Very low	17	0	0	0
Low	8	6	4	7
Medium	14	9	8	13
High	6	16	14	20
Very high (exitus)	4	12	8	20

Supplementary Table S3. Clinicopathological characteristics of hospitalized COVID-19 patients vs serum C5a levels†

	n	C5a (ng/ml)	p value‡
**Age (years)**			
<=65	18	26.7 (17.7-27.8)	0.125
>65	14	31.6 (26.6-36.4)	
**Sex**			
Female	15	26.2 (17.8-27.9)	0.123
Male	17	31.0 (26.1-36.1)	
**Exitus**			
No	28	27.2 (17.6-32.8)	0.072
Yes	4	33.5 (30.6-38.0)	
**Comorbilities**			
No	11	26.2 (17.8-27.7)	0.155
Yes	21	31.0 (21.0-36.1)	
**Type of comorbility**			
Hypertension			
No	17	26.2 (17.5-27.9)	0.082
Yes	15	32.1 (24.1-36.3)	
Diabetes			
No	28	27.4 (17.6-32.9)	0.23
Yes	4	32.9 (29.8-37.1)	
Cardiopathy			
No	20	27.2 (17.9-28.3)	0.182
Yes	12	33.5 (24.0-36.2)	
Kidney disease			
No	28	27.5 (17.9-35.5)	0.978
Yes	4	28.6 (24.0-36.2)	
COPD			
No	27	27.5 (18.8-33.5)	0.880
Yes	5	27.6 (15.5-36.1)	
Immunosupression			
No	28	27.4 (17.9-34.9)	0.491
Yes	4	30.4 (26.4-33.0)	
Cancer			
No	29	27.4 (17.6-34.8)	0.185
Yes	3	31.3 (30.4-34.6)	
Obesity			
Normal	18	27.9 (22.3-34.3)	0.212
Overweight	9	27.6 (17.6-41.3)	
Obese	5	16.2 (14.9-27.1)	
**Symptoms at admission**			
No	7	27.9 (26.8-33.7)	0.395
Yes	25	27.4 (17.5-34.8)	
**Type of symptoms**			
Fever			
No	13	31.0 (27.5-35.3)	0.084
Yes	19	26.2 (16.9-31.3)	
Malaise			
No	22	27.9 (21.3-35.2)	0.325
Yes	10	23.6 (16.5-30.4)	
Arthromyalgia			
No	24	27.9 (19.2-35.5)	0.404
Yes	8	26.8 (17.2-28.5)	
Asthenia			
No	22	27.9 (21.3-35.9)	0.176
Yes	10	23.6 (17.7-30.1)	
Respiratory problems			
No	11	27.6 (23.5-33.7)	0.558
Yes	21	27.1 (17.5-34.8)	
Cough			
No	14	29.3 (21.6-34.5)	0.419
Yes	18	26.7 (17.6-33.4)	
Dyspnea			
No	22	27.4 (17.7-31.9)	0.251
Yes	10	29.5 (26.4-40.0)	
Digestive problems			
No	24	27.5 (17.6-35.1)	0.848
Yes	8	29.4 (19.2-32.9)	
Nausea			
No	25	27.4 (17.5-34.8)	0.679
Yes	6	29.4 (21.6-31.9)	
Unknown	1	36.1	
Diarrhea			
No	24	27.5 (17.6-34.9)	0.982
Yes	7	27.5 (18.8-31.7)	
Unknown	1	36.1	
Neurologic problems			
No	26	28.7 (26.2-35.9)	0.012
Yes	6	17.6 (16.3-17.9)	
Headache			
No	27	27.9 (23.0-35.7)	0.03
Yes	5	17.6 (15.8-18.0)	
Anosmia			
No	30	27.7 (20.0-35.2)	0.226
Yes	2	17.8 (17.7-17.9)	
Ageusia			
No	30	27.7 (20.0-35.2)	0.2226
Yes	2	17.8 (17.7-17.9)	

† In the first sample collected after hospital admision, and expressed as median (25th to 75th percentiles).
‡ Kruskal-Wallis or Mann-Whitney U test.

Supplementary Table S4. Symptoms in discharged COVID-19 patients after a follow-up of two weeks (F14) or three months (F90)†

	F14	F90
**Symptoms**	**n (%)**	**n (%)**
No	15 (60)	20 (83)
Yes	10 (40)	4 (17)
**Type of symptoms**		
Fever		
No	25 (100)	24 (100)
Yes	0 (0)	0 (0)
Malaise		
No	25 (100)	24 (100)
Yes	0 (0)	0 (0)
Arthromyalgia		
No	22 (88)	23 (96)
Yes	3 (12)	1 (4)
Asthenia		
No	17 (68)	20 (83)
Yes	8 (32)	4 (17)
Respiratory problems		
No	21 (79)	23 (93)
Yes	4 (21)	1 (7)
Cough		
No	23 (92)	24 (100)
Yes	2 (8)	0 (0)
Dyspnea		
No	23 (92)	24 (100)
Yes	2 (8)	0 (0)
Digestive problems		
No	24 (96)	24 (100)
Yes	1 (4)	0 (0)
Nausea		
No	24 (96)	24 (100)
Yes	1 (4)	0 (0)
Diarrhea		
No	25 (100)	24 (100)
Yes	0 (0)	0 (0)
Neurologic problems		
No	25 (100)	24 (100)
Yes	0 (0)	0 (0)
Headhache		
No	24 (96)	23 (93)
Yes	1 (4)	1 (7)
Anosmia		
No	25 (100)	24 (100)
Yes	0 (0)	0 (0)
Ageusia		
No	25 (100)	24 (100)
Yes	0 (0)	0 (0)

† From the cohort of hospitalized patients (n=33), symptoms were not recorded from 8 patients at F14 and 9 patients at F90.
